# Supplementary material for: Removal of reinforcement improves instrumental performance in humans by decreasing a general action bias rather than unmasking learnt associations
Source: PLoS Comput Biol. 2022 Dec 8;18(12):e1010201. doi: 10.1371/journal.pcbi.1010201 (PMC9767373; doi:10.1371/journal.pcbi.1010201)
Supplement: Attachment — Submitted filename: ResponseToReviewers.pdf [file pcbi.1010201.s002.pdf]

We would like to thank the reviewers for their detailed and thoughtful comments. They have led to a substantially revised manuscript. The reviewers' comments inspired a set of changes, including changes to the manuscript and further analyses and modeling. We believe that the changes we made in response to the reviewers' comments and suggestions represent a substantial improvement over our initial submission. We are therefore grateful for the time and effort the reviewers put into our paper. We reproduce each of the reviewers' comments below in bold before our response to each point. Changes made to the manuscript are in blue, both here and in the manuscript with highlighted changes.

Please note that, in addition to the responses to each reviewer's specific points, their thoughtful comments also inspired some additional changes which we detail at the end in the section "Further changes".

Reviewer #1

This work investigates the potential performance benefits of removing reinforcement during learning. To do so, the authors subjected 60 healthy volunteers to a novel visual go/nogo learning task with deterministic contingencies, and with blocks with and without feedback (i.e. no reinforcement). Performance was quantified by the sensitivity index d'.
As expected, blocks with no-reinforcement showed improved instrumental performance. However, this improvement was not due to increased sensitivity to learned value (i.e. unmasking task knowledge) but to a reduction in overall propensity to act (i.e. reduced response bias).

This is a very interesting study, which reconciles seemingly contradictory results in humans (omitting feedback impairs learning performance) and animals (omitting feedback improves learning performance). The paper is very clearly written, the hypotheses are very clearly stated, and the methods seem sound – a combination of behavioural experiments and state-of-the-art computational models and modelling procedures (model comparison, parameter recovery, simulation). The findings appear to be supported by the data. My only concern is the interpretation of the results and their generalization to other learning environments, where exploration and risk-taking, for example, may be beneficial.

We thank the reviewer for this very positive overall assessment of our manuscript, but also for suggesting possible alternative interpretations of the data for spotting some points that required clarification.

1. Here, the results suggest that the better performance observed on trials without feedback can be explained by a reduced propensity to act. I wonder to what extent this result is task-specific. Adopting cautious response strategies (that minimize false alarms) may be beneficial in some learning environments, but detrimental in others. For example, in richer or less risky environments, a high propensity to act may be beneficial because the cost of missing an opportunity is greater than the cost of a false alarm. Would you expect the same results with different reward probabilities/magnitudes?

We completely agree with the reviewer. Indeed, we had argued in the discussion of the previous version of this manuscript that the key driver of the behavioural effect is the task asymmetry: It is beneficial to have a high propensity to act because participants gain task information from it (versus no feedback on no-response trials). Therefore, in our setting, an adaptive propensity to act, depending on whether participants can learn from their responses, is beneficial. This is something we now explicitly mention in the discussion (line 379-389):

Finally, these findings hinge on the specific task structure. Our task encouraged an asymmetric response pattern: Performing an action (i.e. go response) is the only possibility to learn and explore possible outcomes for specific stimuli in our task, as refraining from responding yielded no feedback. Therefore, go responses provide a gain in information (information bonus), making them advantageous relative to no-go responses. This information bonus may account for the two main effects our computational model revealed: On the one hand, the information bonus is greatest early in the experiment, because values are not reliable learnt yet. This is reflected in the positive value initialization in our computational model. On the other hand, information can only be obtained in reinforced trials, resulting in a more pronounced general bias to withholding responses in non-reinforced trials.

Furthermore, we think the reviewer raises an interesting point that taps into foraging-related questions, i.e., the role of the average reward rate and resulting opportunity costs. While our data cannot speak to this question (we had a fixed reward magnitude, and reinforcement was fully deterministic), we think it is highly plausible that higher/lower reward probabilities or magnitudes (or any other manipulation that would increase/decrease the average reward rate) would lead to an increased/decreased propensity to act in the reinforced blocks. Indeed, previous work (1) has shown that the average rate of responding is tightly related to the average reward rate in the environment, even when these rewards were not at all contingent on the agent's choices. We have added following paragraph to the discussion about these interesting implications for foraging-related parameters (line 400-403):

Likewise, we assume that an adaptive response bias would also manifest in foraging-related tasks. In previous work, we have shown that the average rate of responding is dependent on the local average reward rate in the environment, even when rewards were not contingent on participants' choices (32).

2. Related to the previous comment: young children sometimes engage in "motor babbling", a self-exploring behaviour that is often described as an excessive propensity for (random) action. This propensity to act has a learning function: it allows the *accidental* discovery of action-effect relationships, which can then be implemented by the child *intentionally*. So I wonder if the benefit of a reduced response bias observed in your task is not due to the fact that the task does not particularly reward this type of self-exploring behavior. Exploration can also be useful in environments with frequent and unpredictable contingency reversals. Would you expect your findings to hold even in environments where exploration is highly advantageous?

This is an interesting point. Especially at the beginning of the task, self-exploring behaviour is rewarded by accidental discovery because participants do not know the value of the choice options. Consequently, they start exploring and have a higher response rate, which is computationally reflected in a positive value initialization. Nevertheless, on average, participants are cautious throughout the task and show a bias towards withholding which is even more pronounced in probe trials, in which self-exploring behaviour is not advantageous. In highly volatile environments (frequent and unpredictable contingency

changes) we would indeed assume the effect of improved performance should be, if anything, more pronounced. Exploration (i.e., responding) would be even more fundamental for learning and for detecting contingency reversals, while this benefit would be absent during blocks without feedback. We have added the following sentence on the possible role of volatility to the discussion (line 403-411):

Furthermore, similar effects may be expected for learning environments characterized by high levels of volatility, where contingencies between choices and outcomes change frequently. In such situations, an agent would benefit from a high response probability on reinforced trials. Conversely, the cost of a false alarm would be greater than the cost of missing an opportunity in non-reinforced trials, resulting in decreased go-response probabilities. Thus, at high levels of environmental volatility, the effect of feedback removal on the response bias might be even more pronounced, as frequent contingency changes further increase the informative value of go-responses during reinforced periods.

3. And more generally: couldn't the apparently contradictory results observed between humans and rodents (improved vs impaired performance when omitting feedback) be due to differences in task environments?

We think the key distinction and putative origin for contradictory results lies in the domain of learning, namely associative versus perceptual learning. In our study, we observed that performance in an associative learning task in humans was improved when omitting feedback, which aligns with the findings in the rodent study that also uses an associative learning task (2). Herzog & Fahle found opposing results in the perceptual domain (3). In contrast to the rodent task and our paradigm, participants could still learn without feedback in this perceptual learning task, nevertheless providing feedback improved the performance further as it simplified the learning process. Therefore, in line with the reviewer's suggestion, we assume that the difference between humans and rodents which we introduced in the beginning of our manuscript is most likely due to the different learning domain (associative vs perceptual) and not because of species differences. This is something we address in the discussion (line 360-366). In addition, we have included a new paragraph to the discussion which explicitly addresses differences between rodent and human studies (line 367-378):

Due to challenges inherent to translational work, one might argue that differences in task environments between the rodent tasks used by Kuchibhotla and colleagues' (12) and our human task could lead to results relying on different mechanisms. For example, extinction is well known to occur when animals' actions are not reinforced (as in the probe trials, 29,30). To prevent extinction effects to confound results, only short probe phases were used in the rodent study (12), making it unlikely that their effects are influenced by extinction. Because rodents did not know that their knowledge is tested in probe trials, we aimed to adapt instructions accordingly. To avoid that participants try to perform better in probe trials, we instructed them to continue responding to the task in non-reinforced trials as they did in reinforced trials.

Reviewer #2

This is an interesting study using a behavioural task in healthy human volunteers and computational modelling. The authors investigate an effect known from rodent studies in which accuracy on an instrumental task increases in blocks in which no feedback is given, compared to blocks in which animals are both making choices and learning the value of items in parallel. The authors replicate this effect in human observers using a go/no-go task. Through careful use of modelling the authors show that improved performance in no-feedback ('probe') blocks is best explained in terms of a reduction in response-willingness, or response bias as they call it. This impacts on both types of active response (hits and false alarms) but it affects false alarms more, because participants have, especially in early blocks of the task, a positive response bias overall (they respond more often than they optimally should).

I thought this was interesting, carefully done and clearly presented. I find no errors per se. My main comments would be about the interpretation – I would like to invite the authors to think about these and comment in the manuscript text, if they find it appropriate to do so.

We thank the reviewer for the positive evaluation of our manuscript, but also for their thoughtful comments.

1) Firstly, I wonder if humans and rodents treat the no-feedback blocks in the same way. In humans, we call them probe blocks as we are testing the observers knowledge, and the observer knows this. Thus we could expect the observers to become more cautious about making errors and try harder in the probe blocks. In contrast, rodents do not understand this demand characteristic – we sometime call no-feedback blocks in rodent tasks testing in extinction' and expect their knowledge of the stimulus-outcome contingencies to decay in this time. Given this conceptual difference how sure can we be that the human task taps the same constructs as even the exact same task done in rodents?

As the reviewer acknowledges, there generally is an inherent difficulty in making sure any task taps into exactly the same construct across species. Nevertheless, we strived to minimize these differences in the following ways: We did not tell our participants that their knowledge is tested during probe trials; We only told them that the reinforcement will disappear from time to time and instructed them to continue responding to the task as they did before (see line 474-476). This mimics the rodent scenario reasonably closely – within the limits possible in humans, of course. Moreover, the extinction process in rodents does not start immediately when reinforcement is omitted as animals need time to realise that their actions are not reinforced anymore. Accordingly, to prevent extinction effects to confound the results, Kuchibhotla and colleagues used only short probe phases (2), suggesting that their effects are not influenced by extinction. We have added the following paragraph to the discussion to clarify this point (line 369-378):

Due to challenges inherent to translational work, one might argue that differences in task environments between the rodent tasks used by Kuchibhotla and colleagues' (12) and our human task could lead to results relying on different mechanisms. For example, extinction is well known to occur when animals' actions are not reinforced (as in the probe trials, 29,30). To prevent extinction effects to confound results, only short probe phases were used in the rodent study (12), making it unlikely that their effects are influenced by extinction. Because rodents did not know that their knowledge is tested in probe trials, we aimed to adapt instructions accordingly. To avoid that participants try to perform better in probe trials, we instructed them to continue responding to the task in non-reinforced trials as they did in reinforced trials.

2) Secondly, I think the effects could be explained in terms of an information bonus associated with active responding (if participants always withhold responding they will never learn which greebles are rewarded) – which is greatest early in the experiment, and absent on probe trials. Maybe the reason for the positive response bias is the information bonus. I think this possibility should at least be addressed in the text (even if the authors have aa counter-argument). If this is the explanation it doesn't diminish the value of the current study but does suggest follow-ups (what would happen if you always showed the counterfactual on non-response trials)?

We completely agree – this is exactly how we think about it and now added this reasoning more explicitly to the manuscript: Responding is advantageous due to the information bonus. This information bonus explains exactly the behaviour we found and which is in line with research about curiosity (4): Uncertainty makes participants curious, thus, they seek for information. On the one hand, this is evident in an initially higher response rate, which, computationally, is reflected in the positive value initialization. On the other hand, participants cannot seek for information in probe trials, which is reflected in a more pronounced general bias towards withholding a response in these trials. In the discussion, we had thus far only mentioned that performing a response is the only possibility to learn, but we have now added a new paragraph to make the explanation of the adaptive response bias more explicit (line 379-391).

Finally, these findings hinge on the specific task structure. Our task encouraged an asymmetric response pattern: Performing an action (i.e. go response) is the only possibility to learn and explore possible outcomes for specific stimuli in our task, as refraining from responding yielded no feedback. Therefore, go responses provide a gain in information (information bonus), making them advantageous relative to no-go responses. This information bonus may account for the two main effects our computational model revealed: On the one hand, the information bonus is greatest early in the experiment, because values are not reliable learnt yet. This is reflected in the positive value initialization in our computational model. On the other hand, information can only be obtained in reinforced trials, resulting in a more pronounced general bias to withholding responses in non-reinforced trials. The resulting behaviour is in line with research about curiosity, suggesting that a lack of information makes individuals curious and facilitates information seeking behaviour (31).

As the reviewer suggested, follow-ups would be possible, e.g., providing counterfactual feedback. This is something we have addressed in the discussion (see line 391-399 and response to Reviewer #1). Given the reviewers' suggestions, we tried to clarify the point in the discussion:

Different tasks that do not encourage go responses for optimal performance are unlikely to result in a beneficial effect of reinforcement removal. Specifically, environments that do not favour any specific response (e.g., situations where the outcomes of both choice options are always presented) will result in a neutral value initialization, because participants do not need to perform a go response in order to gain information. Any change in response bias (if present) would affect hit and false alarm rates to the same extent, thus resulting in no difference in performance. Following the same logic in reverse, environments favouring no-go responses should result in a negative value initialization, thus resulting in the opposite pattern: a reduced performance during non-reinforced trials.

3) Line 133

"We compared performance of the 36 trials in a probe block with performance in the 36 trials before each probe block (pre-probe trials)."

Isn't this confounded with trial number though? There is a general rising trend in d' so always comparing probe with pre-probe (rather than post probe) should lead to probe having a higher d'. I realise people can't be learning during the probe trials but they will he learning during the preceding 30 trials, so the average of 30 pre-probe trials underestimates the instantaneous d' at the start of the probe block. That said I see d' is lower in the post probe trials than the probe trials so I do believe there is a real increase in performance in the probe blocks.

I see later this confound is pointed out and (hopefully) resolved by the introduction of the model. Nevertheless I find it odd to read the first section (pre-model) and not see this confound acknowledged or dealt with. Maybe some rephrasing, or an acknowledgement of the issue and an indication that the model will later address this issue is in order?

The reviewer is completely right and provided a more intuitive summary of the confound than we managed to provide in our previous attempts. This is indeed the same confound we observed and the motivation for introducing the modelling. The reason we presented our

findings in that order was because we wanted it to closely reflect the real progression of our work. At first, we aimed at replicating the rodent findings in humans, following as closely as possible the procedures in the rodent work. It was only then that we realised the confound (which by the way likewise applies to the rodent findings), described it and went on with how we solved it (modelling). We agree however that it will be helpful to introduce this earlier on. As the reviewer suggested, we now restructured the Results section: After explaining the task, we now acknowledge the confound of the signal detection theory-based analyses and introduce computational modelling as a second analysis approach (see section "Analysis approaches: Signal detection theory and computational modelling", line 131-144):

To assess effects of the removal of reinforcement on task performance, we present two approaches. First, we aimed to replicate the results of Kuchibhotla and colleagues' work (12) based on signal detection theory (SDT). To this end, we computed the sensitivity index *d'*, representing the difference in the relative frequencies of hits and false alarms (button presses to go and no-go stimuli, respectively, 16). To compute measures from SDT, it is necessary to consider windows of several trials. Importantly, this approach can introduce artefacts in learning paradigms, due to its insensitivity to the general rising trend in performance. Specifically, during learning mean performance on earlier trials is intrinsically lower than mean performance on later trials, irrespective of any manipulation, such as feedback removal. Consequently, *d'* implicitly disadvantages earlier trials compared to later ones, unless performance has reached stable levels. For this reason, we next present a computational modelling approach, which averts this issue by providing trial-by-trial estimates. Conclusions are primarily based on this second approach, presented in the paragraph "Computational modelling reveals a shift in action bias".

Then we go on with presenting the replication of the rodent work (section "Sensitivity index *d'* increased when reinforcement is omitted"), followed by the modelling results (section "Computational modelling reveals a shift in action bias").

4) Line 214

"On probe trials in which the go action was selected (and no feedback received), no changes were applied to value estimates. During trials in both reinforced and probe blocks without a go action, we assumed that values were subject to passive forgetting (21,22) via diffusion towards zero governed by the decay parameter θ."

Why are no-feedback go trials not subject to decay?

There were two reasons for this, one theoretically, and one empirically grounded. The non-monotonic plasticity hypothesis (5) states that low to intermediate activation of an association will cause it to weaken, while high levels of activation will cause a strengthening. On the basis of this, we had previously hypothesized that presentation of a stimulus, without it being actively chosen, will lead to moderate activation of the retrieved association, while actively choosing it will lead to strong activation - both in the absence of feedback on the choice. This is exactly what we found, both at the behavioural (increased preference for chosen, decreased preference for unchosen items) and neural level (signatures of increasing/decreasing stimulus-outcome associations in the hippocampus) (6). Therefore, we assumed in our setup that values in no-feedback trials should not be subject to decay, when participants performed a response.

Notwithstanding these considerations, we tested the effect of also applying the decay to the go trials without feedback (response trials in the probe blocks), as suggested by the reviewer. We implemented this for the baseline model to test whether this additional decay would increase or decrease model fits. We found that this model variant performed worse compared to our previous model which only considers no-response trials for decay ($BIC_{nofeedback}$ = 600.70 ± 117.24, mean ± SEM, $BIC_{baseline}$ = 539.42 ± 137.39) and this model could not qualitatively reproduce the behavioural pattern (see Fig I). We included this in the supplements (see Figs 10D and 11B in S1 Appendix, reproduced below in Fig I).

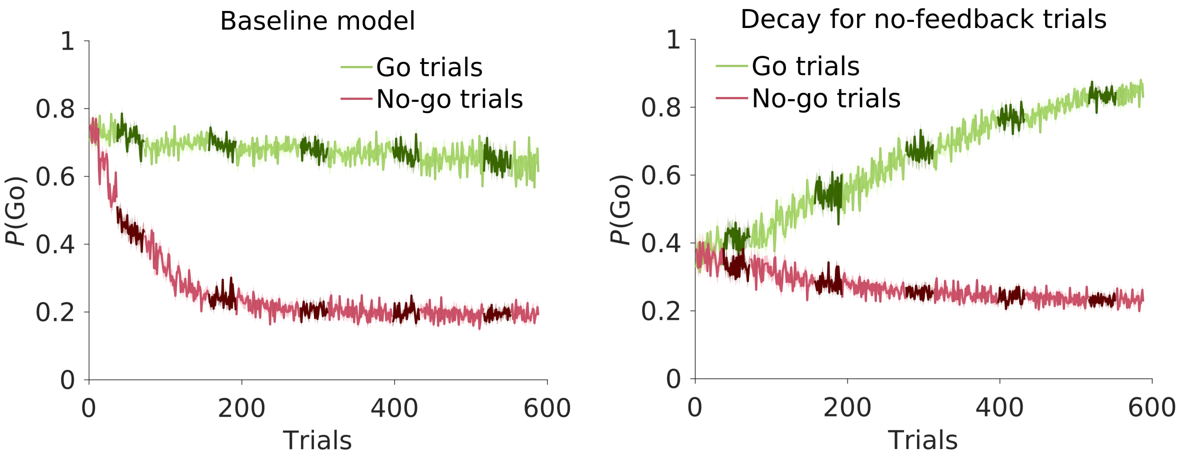

Fig I. Different implementations of passive decay in the baseline model.
Time course of simulated go-response probabilities, *P*(Go), for go trials (green) and no-go trials (red). Darker shades of green and red illustrate probe trials. Solid lines represent mean, shaded areas SEM across simulations. Simulations are based on the baseline model (including a softmax temperature, a general bias, an initial *Q*-value and a decay parameter). Left: Baseline model as used in the manuscript. No-response trials were considered for

decay. Right: Decay implemented for all trials without feedback, including go-responses in probe trials.

5) Line 234 f3c

Initial value estimates are optimistic. Conceptually, could this reflect an information bonus rather than an optimistic value estimate per se? Optimistic value estimate means (I think) that participants respond 'go' more often than they should. But they only find out the value of the greeble (whether positive or negative) if they respond – withholding the response = no information.

Indeed, we believe that this optimistic value estimate essentially is a result of the information bonus and pointed this out in the same section where we have discussed the information bonus (see above). Furthermore, we think that optimistic might be misleading in this context, we have therefore changed this accordingly to 'positive value initialization' in the manuscript (lines 260, 263, 304, 317).

6) Furthermore, the information bonus should be absent in probe trials, perhaps this could drive the reduced (overall) tendency to respond in probe trials

Yes, we suspect that the information bonus of go-responses drives the positive value initialization and reduced response rate in probe trials, too. We described it in more detail in the previous comment 2 and added a paragraph in the discussion (line 379-391):

Our task encouraged an asymmetric response pattern: Performing an action (i.e. go response) is the only possibility to learn and explore possible outcomes for specific stimuli in our task, as refraining from responding yielded no feedback. Therefore, go responses provide a gain in information (information bonus), making them advantageous relative to no-go responses. This information bonus may account for the two main effects our computational model revealed: On the one hand, the information bonus is greatest early in the experiment, because values are not reliable learnt yet. This is reflected in the positive value initialization in our computational model. On the other hand, information can only be obtained in reinforced trials, resulting in a more pronounced general bias to withholding responses in non-reinforced trials. The resulting behaviour is in line with research about

curiosity, suggesting that a lack of information makes individuals curious and facilitates information seeking behaviour (31).

Reviewer #3

In this study, the authors investigate human reinforcement learning using a Go/No-Go deterministic task performed by a total of 60 participants over 2 experiments, in an attempt to replicate previous results recently found in rodents (Kuchibhotla et al. Nat Commun 2019). The results from the original study were partly replicated in humans: when comparing blocks without feedback ('probe' trials) to blocks where feedback was provided ('reinforced' trials), the measured sensitivity increased. This was due to a decrease of both the false alarm rate (button press in No-Go trials) and the hit rate (button press in Go trials), the latter effect not having been found in rodents. Next, the authors turn to computational modeling and elegantly show that the behavioral effects result from a shift in the action bias in probe trials, rather than an increased sensitivity to the learnt option values. Overall, the paper is clear and well written. This work represents an important and relevant research topic in the field, however I have some concerns regarding the behavioral interpretations and modeling procedures that I think the authors should address.

We are very happy about this positive overall assessment and we thank the reviewer for their thoughtful comments.

Behavioral results

1) One major concern pertains to the definition of performance. In this task, a button press is rewarded in Go trials, while it is punished in No-Go trials. Therefore, an accurate measure of the performance would be the proportion of button presses in Go trials and withholding in No-Go trials, yet in the paper the 'performance' is approximated by the sensitivity d', which is the difference between normalized hit rate (HR) and false alarm rate (FAR). In the original study, the increased sensitivity relied on no change in the HR but a decrease in FAR (hence, increased performance). In this

study however, both HR and FAR decrease, which means that the performance increases in No-Go trials BUT decreases in Go trials. At best, this should be addressed and discussed in the manuscript; at least, the manuscript will benefit from displaying the actual performance changes between probe and reinforced trial blocks.

We agree with the reviewer that by 'performance', it would be more natural to think about accuracy, i.e. the percentage of correct choices (choice probabilities) for either the go- or no-go-options. The reason for primarily focusing on *d'* (and HR and FAR) in the first place was that we wanted to closely follow the procedures adopted by Kuchibhotla and colleagues in their rodent work (2). Indeed, the sensitivity index *d'* captures to what extent participants are able to discriminate between the two stimulus types (go and no-go). The reviewer is correct that our results diverge slightly from the rodent findings in that, for them, only the HR decreased in probe trials. We now acknowledge this discrepancy to our findings (line 154-159):

This increase in *d'* was driven by a more pronounced reduction of false alarm rate (*FAR*) than hit rate (*HR*; $\Delta FAR - \Delta HR = -0.07 \pm 0.08$, $t_{59} = -6.42$, $p < .001$, Cohen's $d = -0.83$). However, both measures decreased significantly in probe compared to pre-probe trials ($\Delta HR = -0.08 \pm 0.06$, $t_{59} = -9.67$, $p < .001$, Cohen's $d = -1.25$; $\Delta FAR = -0.15 \pm 0.08$, $t_{59} = -15.12$, $p < .001$, Cohen's $d = -1.95$, Fig 2C/D), while in (12) only a decrease in false alarm rate during probe trials was reported.

We agree that *d'* is not a measure of performance that is straightforward to interpret, so we now included the choice probabilities for both go and no-go trials as these provide a more intuitive metric of performance (and a measure that is closer to the raw data), allowing comparison of learning specific to go and no-go trials. This is now included in the main figures Fig 1C and Fig 3E (reproduced below). Please note, however, that the same confound with the trial number that applies to *d'* likewise exists with the choice probability data (albeit less pronounced as shorter windows of trials are required here).

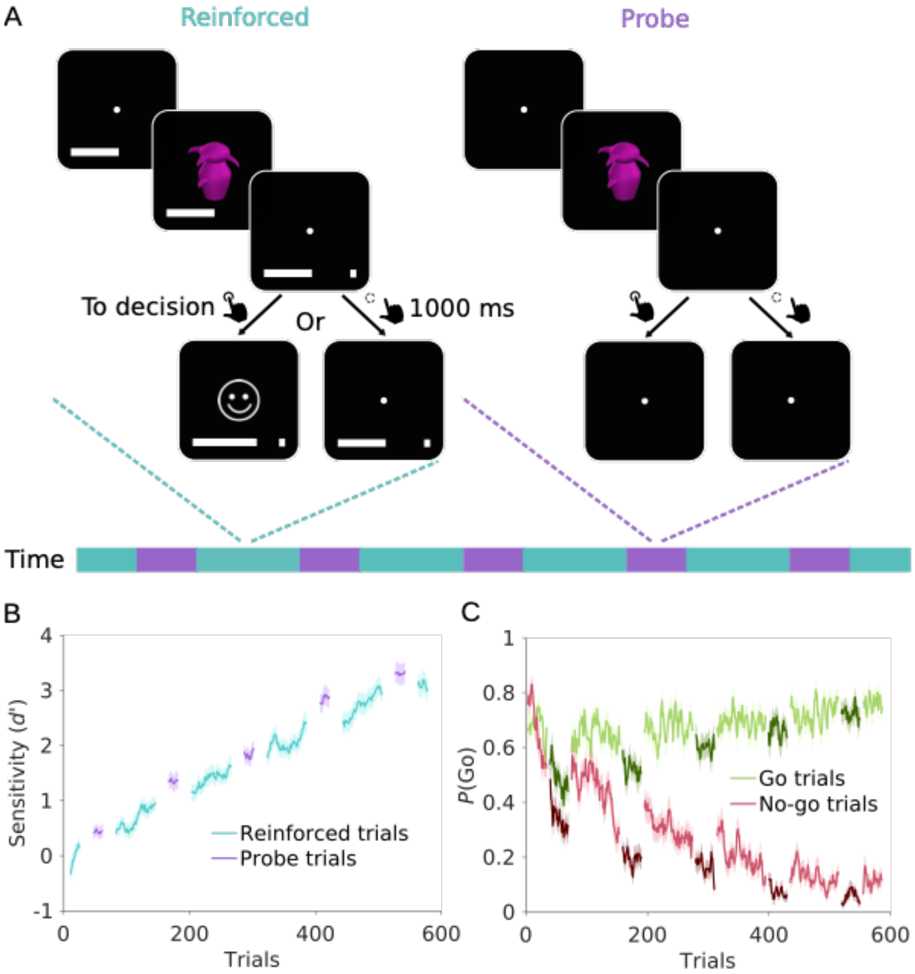

Fig 1. Task structure and participants' behaviour.
(…) (B) Sensitivity index *d'*, separately for reinforced (cyan) and probe trials (purple). (C) Time course of go-response probabilities, *P*(Go), for go trials (green) and no-go trials (red). Darker shades of green and red indicate probe trials. Solid lines in B and C represent mean, shaded areas SEM across participants.

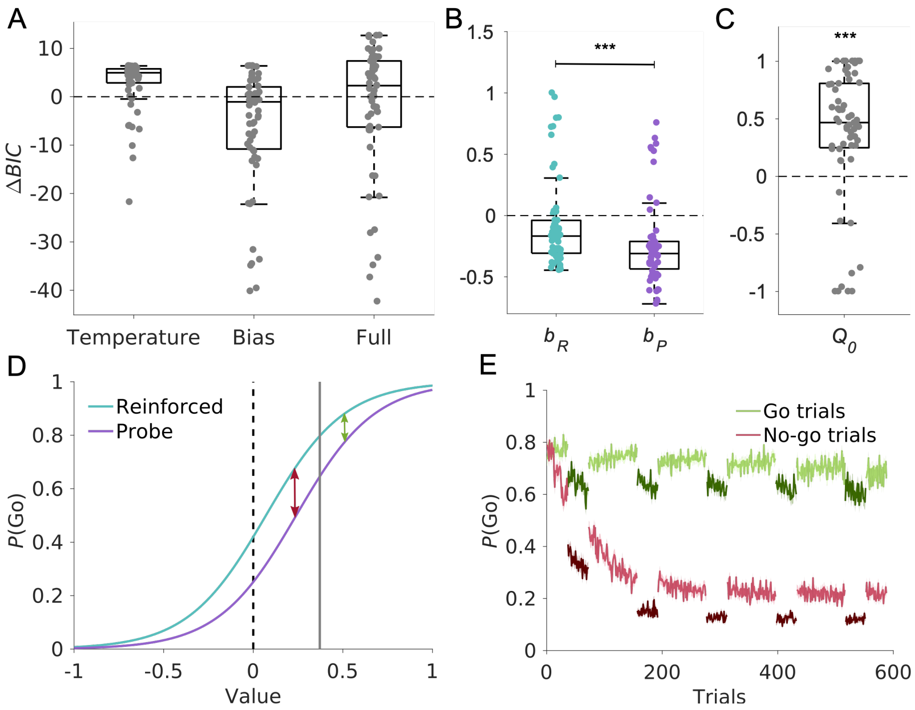

Fig 3. Computational modelling results.
(…) (E) Time course of simulated go-response probabilities. The probability *P*(Go) for go trials (green) and no-go trials (red) was simulated based on the bias model. Darker shades of green and red indicate probe trials. Solid lines represent mean, shaded areas SEM across simulations.

2) One of the key results from the original study was that not only does the FAR decreased during probe blocks compared to pre-probe blocks, but it also increased again in post-probe blocks. This result is partly replicated here, since it was only the case in one of two experiments. I believe this should be mentioned in the manuscript (lines 141, 189-190), especially since the authors chose to present pooled results for both experiments (even though some results do not replicate). Yet, I believe that presenting the results of both experiments separately in the supplementary materials is good practice and should be encouraged.

True, this is indeed a difference we should mention in the manuscript. We have now inserted this in the results (see line 159-162), reproduced below in blue:

Once reinforcement was reinstated, *d'* significantly decreased again ($\Delta d'$ = 0.15 ± 0.47, t_{59} = 2.40, *p* = .020, Cohen's *d* = 0.31, see Fig 7 in S1 Appendix). Note that this effect was only evident in Experiment 2 when analysing the two experiments separately (see Section 1 in S1 Appendix).

Computational results

3) My main remark concerning the modeling part of the manuscript, is that the results seem to come as a surprise, while they could have easily been expected from the behavioral results. This partly comes from the definition of the sensitivity as being a measure of performance (see point 1): based on the model description, increasing the temperature would make the choice more stochastic, i.e., affect the behavior in Go and No-Go trials in opposite directions (decrease p(hit) in Go trials, but increase p(false alarm) in No-Go trials). This would indeed affect the performance but not the sensitivity. To modify the behavior towards more withholding, the simplest way is to add a shift in the decision function (not to modify stochasticity), i.e., a bias towards not responding, as evidenced by model fitting.

We agree with the reviewer here that an increase in temperature will clearly affect *P*(hit) and *P*(false alarm) in opposite direction. However, we argue that an increase in temperature will also affect the sensitivity index *d'*, as this is computed as *d' = z(HR) - z(FAR)*. Thus, when the hit rate *decreases* and, at the same time, the false alarm rate *increases* (as correctly pointed out by the reviewer), this will lead to a smaller *d'*. Exactly this had also been our reasoning to use temperature as readout for how sensitive participants are to acquired values - we had hypothesized that temperatures would decrease during probe blocks, which thus would result in an increased *d'*. Behaviourally, we saw that participants had a reduced response rate in probe trials, but for us it was not obvious that withholding responses could result in an increased *d’* as it requires a stronger reduction in false alarm rates than in hit rates. With that in mind, the computational results came as a surprise for us which is why we investigated how the bias model is able to replicate the behavioural results – not only the withholding, but also the differential decrease in hit rate and false alarm rate.
Thus, we agree with the reviewer that a shift in the decision function (bias) is the easiest way to obtain more cautious responding. Nevertheless, when we inspected (see Fig II) the effects of changing either the temperature (left) or bias (right) in the probe phase, it became clear to us that a simple change in bias would reduce both *P*(hit) and *P*(false alarms) in a symmetric fashion and thus would not change *d'*. Indeed, the change in response bias can only lead to the observed differential effect on hit and false alarm rates when a positive initial estimate for *Q*-values is taken into account. We hope this latter point will also become clearer in response to your next comment.

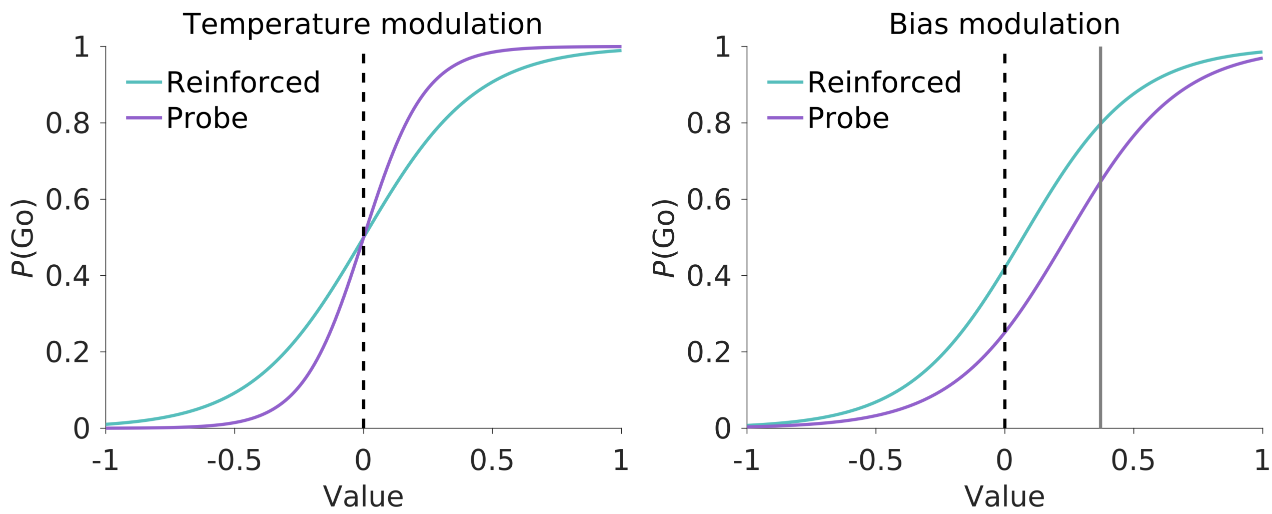

Fig II. Softmax probabilities to perform a go-response as a function of the option's value.

Left: The sigmoids for reinforced and probe trials were generated using example parameters for a reduced temperature in probe trials. Right: The sigmoids for reinforced and probe trials were generated using the mean fitted parameters based on the bias model. Solid vertical grey line indicates average Q_0.

4) Fig 3: related to the previous point, this might change with the Q-value initialization. It seems a bit confusing to have a positive Q0 (i.e., a bias towards acting) AND a negative bias parameter (i.e., a bias towards withholding). Have the authors looked into the possible correlations between parameters? Or parameter values when Q0 is fixed at 0 (i.e., no initial bias)?

This is an important point. Overall, participants are rather cautious, which is reflected in a negative bias parameter (a general propensity to withhold responding). However, there is an information bonus for performing the go response (as this is the only way to obtain information) which impacts particularly the early trials of the experiment when participants still have not learnt anything about the options. Therefore, the two parameters – positive value initialization *and* a negative bias - indeed act in opposite direction. However, Q(0) only sets the starting point (thereby providing an offset to the decision function, see plot above in response to the previous comment) and is modified by learning, whereas the bias parameter captures an average, overall propensity to act across all of the (reinforced or probe) trials. This means that participants start with a bias towards acting which vanishes during the experiment, such that the bias towards withholding occurs. Importantly, as described in the text (and illustrated in Fig 3D), it is the positive value initialization that allows the change in the bias parameter to have an *asymmetric* effect on hit and false alarm rates: A decrease in the bias parameter causes both hit and false alarm rates to decline, but more strongly so for

the false alarm rate. To provide more formal evidence in support of this reasoning, we also ran a variation of the baseline model where *Q*(0), rather than being a free parameter, was fixed at 0, as suggested by the reviewer. We find that this model (i) performs worse compared to our baseline model that incorporates *Q*(0) as a free parameter ($BIC_{Q(0)=0}$ = 591.77 ± 123.25, mean ± SEM, $BIC_{baseline}$ = 539.42 ± 137.39) and (ii) fails to qualitatively reproduce the key behavioural pattern (see Fig III). We have included this in the supplements (see Figs 10C and 11B in S1 Appendix, reproduced below in Fig III).

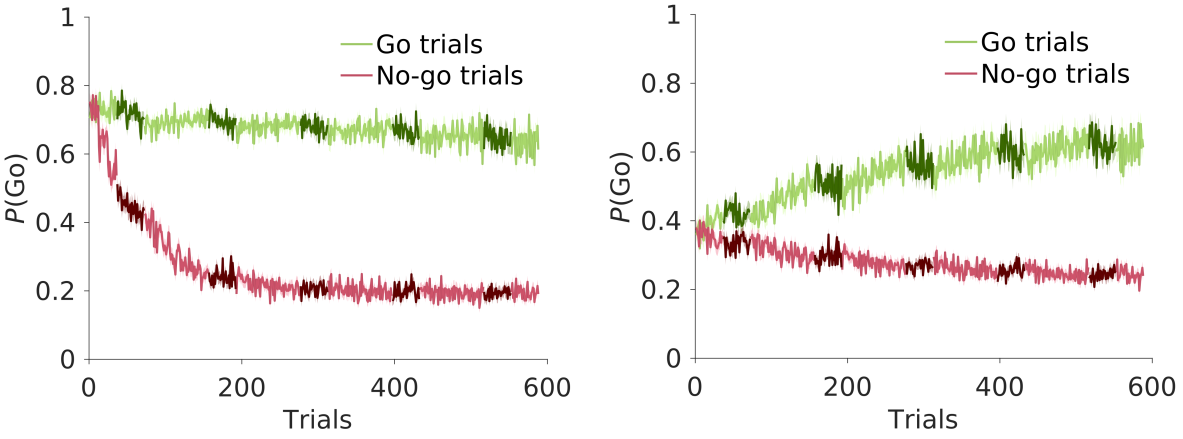

Fig III. Effect of initial Q-value in the baseline model.
Time course of simulated go-response probabilities, *P*(Go), for go trials (green) and no-go trials (red). Darker shades of green and red indicate probe trials. Solid lines represent mean, shaded areas SEM across simulations. Simulations are generated using the baseline model, including a softmax temperature, a general bias and a decay parameter Left: Effect of adding the initial *Q*-value *Q*(0) as additional free parameter. Right: Effect of fixing the initial *Q*-value at *Q*(0) = 0.

5) Line 269: based on Fig 1, I wouldn't say that the model accurately captures the behavioral patterns: the plateau seems to be achieved much sooner. One explanation could be the fixed value which was chosen for the learning rate: although it makes sense to fix that value in the context of this task, it is not clear from the methods, nor from the results, why the value of 0.06 was chosen (since almost all values from tables S11 and S12 seem to produce similar behavioral patterns). Relatedly, the manuscript could benefit from simulations from other models, in order to grasp why the parameters of the winning were needed to produce such behavioral patterns (for reference, see Palminteri et al. TICS 2017). For example, while I agree with the statement on lines 285-287, it will be useful to see that the other proposed models don't achieve this (if, indeed, they don't).

We agree with the reviewer that the shape of participants learning curve is somewhat more reminiscent of a linear progression, rather than the logarithmic convergence of an RL model. The fixed learning rate of 0.06 was an arbitrary choice based on common values for learning rates in reinforcement learning tasks. A smaller learning rate would indeed achieve a better fit to the data, nevertheless, it would not change our main finding: Across the entire range of learning rates tested, the bias model (which combines a reduced general bias in probe trials with a positive value initialization) is the best fitting model, and it consistently performs better than the temperature model, which fits independent temperatures for reinforced and probe trials. We have now included this information in S1 Appendix in Fig 8 (reproduced below) and as Table 11.

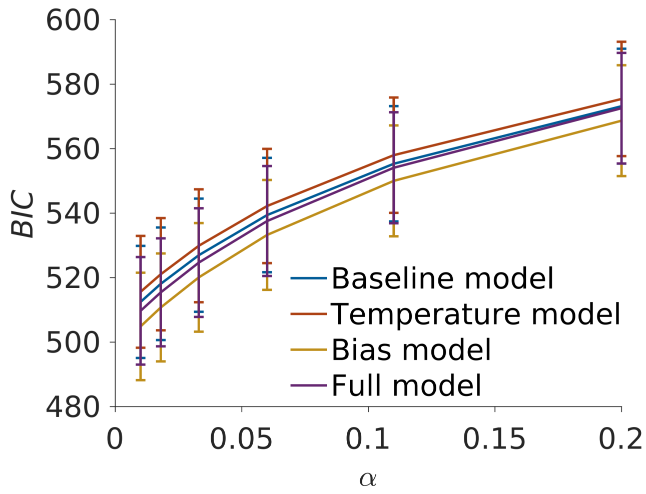

Fig 8. Model comparison with different learning rates.
Comparison of mean *BIC*s for six different learning rates, error bars represent SEM. For all learning rates, the bias model provided the best fit. The full model fitted second best, followed by the baseline model. The temperature model performed the worst.

When looking at the choice probabilities it becomes clearer that the bias model using a learning rate of 0.06 captures key aspects of behaviour – simulated choice probabilities are initialized at a similar level as participants' initial choice probabilities and while the choice probability in go trials stays at this level, the probability for no-go trials decreases. Additionally, there is a decrease in choice probability in probe compared to reinforced trials for both go and no-go trials. We now show in the supplements that all parameters of the bias model are needed to reproduce the behavioural patterns (see Fig 10 in S1 Appendix). We also show the simulated behaviour of the baseline and temperature model which cannot capture the behavioural patterns (see Figs 10D and 12 in S1 Appendix, reproduced below in Fig IV).

We would like to thank the reviewer particularly for this comment as this has made us rethink our model validation approach and we now use choice probabilities to compare empirical and simulated behaviour (see "further changes", point 1 at the end of this document).

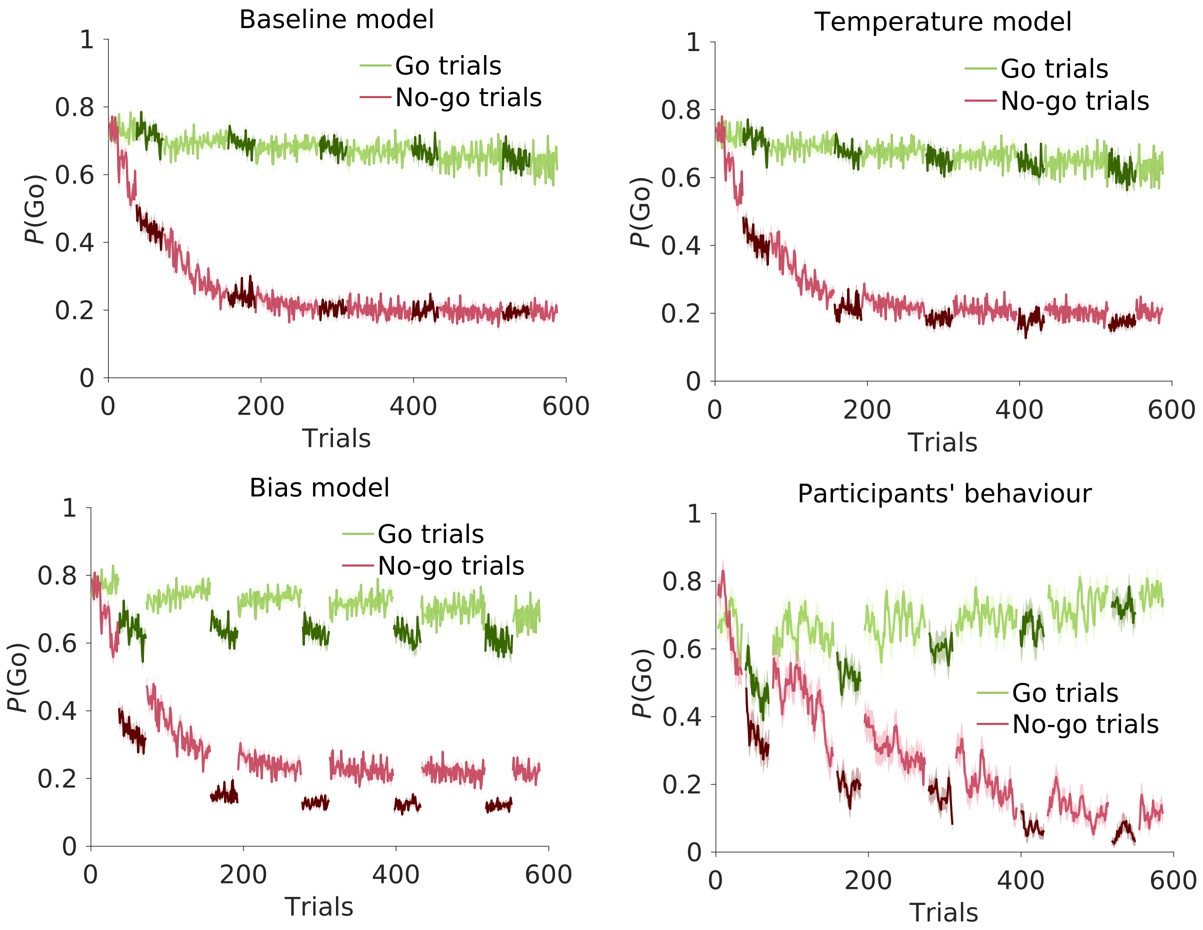

Fig IV. Simulated and empirical go-response probabilities.
Time course of go-response probabilities, *P*(Go), for go trials (green) and no-go trials (red). Darker shades of green and red indicate probe trials. Solid lines represent mean, shaded areas SEM across simulations.

6) Line 506: it is not clear from the methods whether this passive forgetting is performed on the Q-value of the displayed option, or all others as well. In which case, (1) this could be extended to all trials (including Go trials) and (2) the weakening of value associations can also be achieved with a forgetting rule such as those from Collins and Frank, Eur J Neuro 2012, where the unseen options decay towards their initial value (if the initial Q-value differs from 0, why would forgetting diffuse towards zero?).

Thank you for pointing this out. In the description of this parameter, we have now added that the decay only applies for the displayed option (see line 522), reproduced in blue:

Therefore, on trials during which participants performed a no-go response, we assumed passive forgetting of the displayed option governed by a decay parameter θ (for reinforced and probe trials).

(1) Just as for no-go trials, the passive forgetting applies to go trials, when they are not chosen. Adding passive forgetting for all trials would result in two simultaneous updates for chosen trials - a passive decay and an active update based on the prediction error -, which, in our view, would not seem plausible.

(2) To us it seemed plausible to implement a decay independent of the initial *Q*-value, because the *Q*-value only reflects the high response rate in the beginning of the experiment. When participants forget about a stimulus, they should have a neutral value for this option instead of a positive value. Nevertheless, we ran a variation of the baseline model featuring a decay towards the initial value, as suggested by the reviewer. We find that this model performs worse compared to our baseline model that decays towards 0 ($BIC_{q\text{->}Q(0)}$ = 547.74 ± 116.46, mean ± SEM, $BIC_{baseline}$ = 539.42 ± 137.39) and (ii) fails to qualitatively reproduce the key behavioural pattern (see Fig V). We have included this in the supplements (see Figs 10D and 11A in S1 Appendix, reproduced below in Fig V).

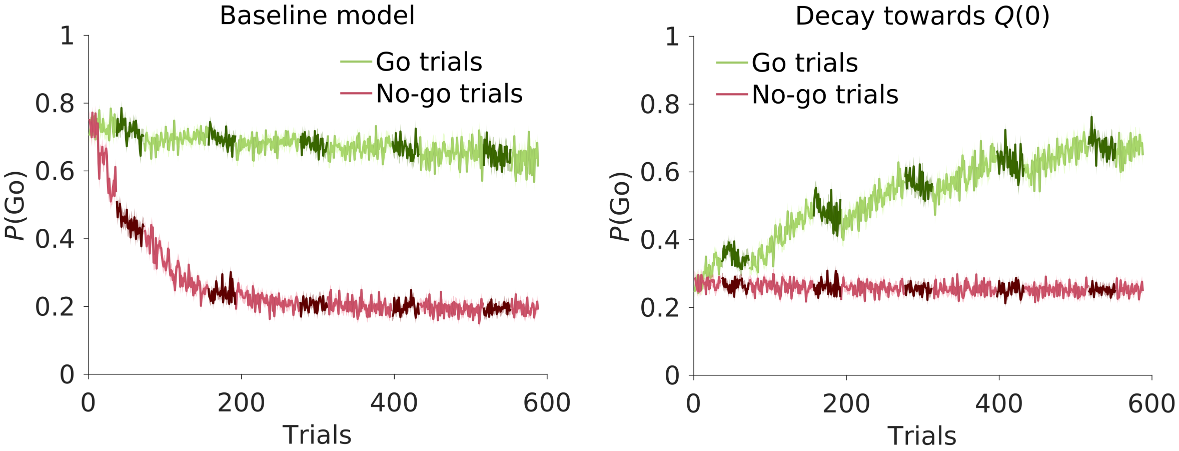

Fig V. Different implementations of passive decay in the baseline model.
Time course of simulated go-response probabilities, *P*(Go), for go trials (green) and no-go trials (red). Darker shades of green and red indicate probe trials. Solid lines represent mean, shaded areas SEM across simulations. Simulations are based on a model including a softmax temperature, a general bias, an initial *Q*-value and a decay parameter. Left: Baseline model with values decaying towards zero. Right: Model with values decaying towards the initial *Q*-value *Q(0)*.

Methods

7) I have some concerns about the inclusion/exclusion criteria. Not only are the criteria defined on the one dependent variable of interest (i.e., the 'performance', which, again, is a measure of sensitivity and not formally task performance), but it led to the exclusion of 34% and 23% of participants in each experiment, which is a very high relative number of rejections. How do the results of the whole dataset look like? Why did the authors choose to stop at 30 participants per experiment? Did the sample of 30 participants reach the BF threshold? In which direction?

To ensure that participants learned to distinguish between go and no-go trials, we used the sensitivity index as exclusion criterion as it exactly measures this aspect. Even though the exclusion criteria as well as the effect of interest both rely on the calculation of *d'*, we do not think this poses a problem, because for the exclusion criterion we calculated *d'* across all trials of the second half of the task, that is, not making the distinction in reinforced and probe trials. Therefore, we consider the exclusion of participants independent from the effect of interest.

The stopping rule was based on included participants only. Before we started the experiment, we defined the sampling procedure as follows: (1) In any case, we would acquire at least 30 participants. (2) Once those 30 were reached, we would continue recruiting as many participants as necessary to reach a pre-defined *BF* that either provides strong evidence in favour of our hypothesis (BF_{10} > 10) or strong evidence against it (BF_{01} > 10) (7). As the *BF* exceeded 10 already after 30 participants, we did not continue sampling more participants. We acknowledge that this information was not given in the manuscript so far and added it in the methods (see lines 437-440).

For both experiments, we found strong evidence in favour of an overall change in *d'* between reinforced and probe trials after inclusion of 30 participants, such that data acquisition in both experiments was stopped after 30 included participants.

We used 12 different stimuli to make the task difficult and thus obtain slow, gradual learning. This resulted in the high number of rejections. Some people failed to learn altogether or learned too slowly (see Fig VI below). Therefore, we do not think they can be used to make inference about our research question. If the reviewer thinks this it would be helpful to include this information in the supplements, we would of course be happy to do so.

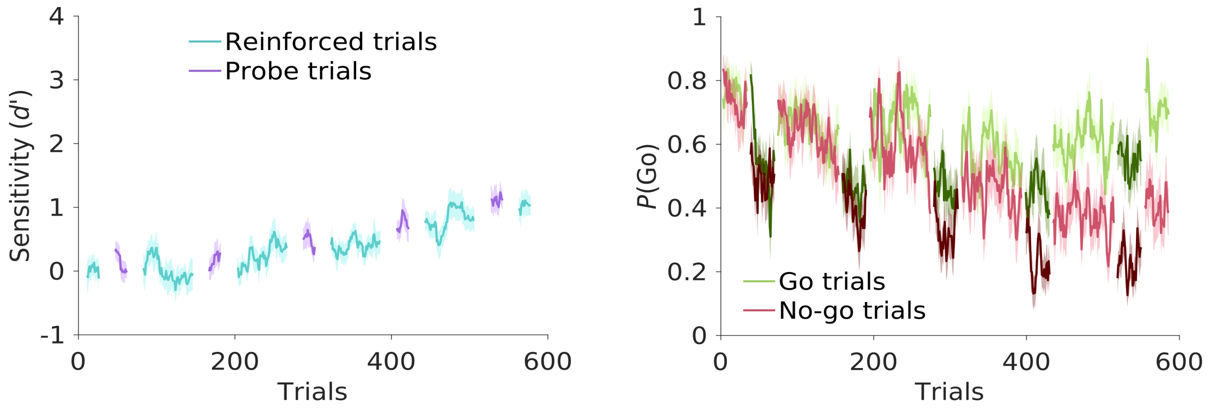

Fig VI. Behaviour of excluded participants (*N* = 25).
Left: Participants' learning curve. The sensitivity index *d'* was separately computed for reinforced (cyan) and probe trials (purple). Solid lines represent mean, shaded areas SEM across participants. Right: Time course of go-response probabilities, *P*(Go), for go trials (green) and no-go trials (red). Darker shades of green and red illustrate probe trials. Solid lines represent mean, shaded areas SEM across participants.

8) In this task, the stimuli were presented for 500ms, and participants had 1000ms to either press a button or withhold from doing so. The time window is shorter than those used in similar tasks (e.g., Guitart-Masip et al. Neuroimage 2012, and following studies, where the total reaches 2500ms). Could a different time window have influenced the results (e.g., no action bias towards button press)?

A longer presentation time/response time does not change the asymmetry of the task, i.e., participants cannot learn when not to press a button. From that point of view, it should not influence the results. If participants needed more time to remember, many no-responses should be evident, which would manifest in a cutoff of the RT distribution. The task by Guitart-Masip and colleagues (8), while also being a go/no-go task, is much more complex than ours. In their task, a cue was presented giving information about the action and the outcome valence. Then, participants needed to detect a target which followed the cue after a delay, after which they were asked to give a response or withhold responding. Thus, in addition to detecting the target, the participant also needed to decide based on the cue what action was required and whether this action would be rewarded or punished. Our task is much simpler: On each trial, a single stimulus is presented at the centre of the screen which, if chosen is deterministically followed by either reward or punishment (depending on the stimulus). Therefore, less time is required to make a decision, which can be seen in the distribution of response times which are all well within the response window, as reproduced

below. We added this figure in S1 Appendix as Fig 13 and added the following sentence to the methods (line 463-464):

The response could be administered during stimulus presentation and within 1000 ms after stimulus offset. The duration of this response window was well sufficient to perform a go-response (see Fig 13 in S1 Appendix).

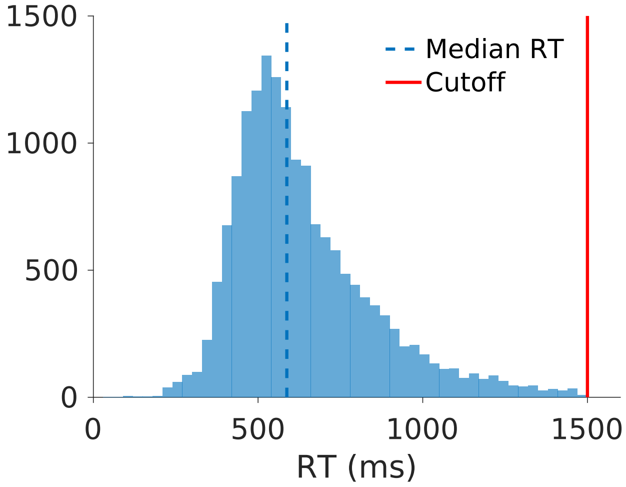

Fig 13. Distribution of participants' RTs.
The cutoff of 1500 ms relative to stimulus onset marks the end of the response window.

Minor points

9) Line 199: I suggest not to mention 'value differences' in this case, since the decision is based on the value of one option only.

Thank you, we changed it (see line 213-216):

At higher temperatures, participants' choices are increasingly stochastic, and large values ~~differences~~ are required to select the correct choice with high probability, while at low softmax temperatures, values slightly greater/lower than zero are sufficient to reliably select the go/no-go action, respectively.

10) Line 202: in general, the temperature should not be perceived as a measure of performance, since in multi-alternative decisions, it rather balances between

exploration and exploitation based on the knowledge at trial t (i.e., if the values are not accurately represented, a lower temperature might lead to more errors).

True, in particular in multi-attribute settings and/or learning scenarios, the temperature governs the balance between exploration and exploitation. It does however index the sensitivity of decision making to acquired values - whether these values are accurately represented or not. We changed the expression ‘index of performance’ to ‘index of choice sensitivity’ and included the following sentence to the section "Computational modelling reveals a shift in action bias" (line 216-219):

While in multi-alternative decisions, the temperature governs the balance between exploration and exploitation, in our paradigm with a single option per trial and deterministic reinforcement, the temperature can be used as an index of choice sensitivity.

11) Line 315: the use of the term ‘optimistic’ might be confusing here, since it is also used in other reinforcement learning tasks as the difference in learning rates from positive or negative prediction errors, not the initial option value or bias towards acting.

Thank you for pointing it out, we changed ‘optimistic value initialization’ to ‘positive value initialization’ in the whole manuscript (lines 260, 263, 304, 317).

12) Line 364: post-learning phases do not necessarily happen on a separate day in human reinforcement learning (see e.g., Frank et al. Science 2004 and following studies; Palminteri et al. Nat. Commun. 2015 and following studies).

True, we changed it now (see line 329-330), reproduced below in blue:

To disentangle these two concepts, the phases designed to assess learning versus expression of task performance are usually separated by a considerable delay in these studies ~~(typically on a separate day)~~.

13) Line 403-405: this is a surprising assumption. Based on behavioral and modeling results, the performance, i.e., withholding from button press, should increase in No-Go trials (regardless of whether there are other types of trials). It is also unclear why the first trial option value should be different from the one in a task featuring both trial types.

In our task, we assume that participants have an initial go bias to gain information. When both go and no-go trials are reinforced, there is no need to press the button in order to gain information and thus we would expect no initial go bias. Based on the softmax function, a simple response bias should not result in a differential decrease for go and no-go trials, when participants start with *Q(0)* = 0 (see our response to point 3 above). To clarify this, we changed it in the discussion (line 391-399).

Different tasks that do not encourage go responses for optimal performance are unlikely to result in a beneficial effect of reinforcement removal. Specifically, environments that do not favour any specific response (e.g., situations where the outcomes of both choice options are always presented) will result in a neutral value initialization, because participants do not need to perform a go response in order to gain information. Any change in response bias (if present) would affect hit and false alarm rates to the same extent, thus resulting in no difference in performance. Following the same logic in reverse, environments favouring no-go responses should result in a negative value initialization, thus resulting in the opposite pattern: a reduced performance during non-reinforced trials.

14) Line 473: the correction for extreme HR is usually based on the number of corresponding trials only (see Macmillan and Creelman 2004, and the original publication), although this might be of little relevance here.

We noticed we have used a different equation for the correction than we have indicated in the methods; in fact, we used $HR = \frac{1}{2N}$ as correction for HR = 0 with *N* being the number of *all* trials. As the reviewer points out, the correction for extreme HR is usually based on the number of *corresponding* trials only. Because we calculated *d'* for a small set of trials, the resulting *d'* would most likely be underestimated, since extreme values cannot be reached with this correction (9). Therefore, we decided to use a correction allowing for extreme values. Please note that the conclusions remain unchanged, irrespective of the specific

choice of correction (*d'* significantly increases in probe trials, $\Delta d'$ = 0.28 ± 0.29, mean ± SEM, t_{59} = 7.53, p < .001, Cohen's d = 0.97). In the methods, we now acknowledge that we did not use the corresponding trials only (see line 484-488):

As ceiling performances cannot be *z*-scored, we used $HR = 1 - \frac{1}{2\mathrm{N}}$ as correction for *HR = 1*, and $HR = \frac{1}{2\mathrm{N}}$ as correction for *HR = 0*, with the number of trials *N*. Because we calculated *d'* for a small number of trials, the use of corresponding trials for correction may result in underestimated *d'* values (34), so we used a correction allowing *HR* and *FAR* to be approximately 0 or 1.

Further changes

Some of the reviewer comments made us rethink certain aspects of our analyses. In the following, we describe a set of changes that were applied to the manuscript, which were triggered by the reviewers' constructive comments, but which are not (directly) related to their specific comments addressed above.

1) Discussion

The reviewer comments inspired us to make the discussion more comprehensive. In order to be able to incorporate all points in a coherent way, we have restructured it entirely.

2) Model validation

In the course of responding to reviewer comments, we realised that using the comparison of pre-probe versus probe trials for model validation is not meaningful, because every model which implements learning could reproduce this artefactual improvement, for the very reasons as the confound related to *d'*. Therefore, in addition to model comparison via *BIC*, we used the choice probabilities for go and no-go trials (as proposed by Reviewer #3) to inspect and compare the empirical and simulated behaviour of the winning bias model. Additionally, we inspected the baseline and temperature model to validate the outcomes of the *BIC*. We found that only the bias model was able to qualitatively recapitulate the

behavioural pattern. We have changed the section “Model validation” in the methods and the following paragraph in the manuscript (line 288-297):

Next, we validated the winning model. Due to the confound in the SDT-based analyses of the behavioural data, any model which includes learning generates a difference between pre-probe and probe trials, hence, examining qualitative difference between reinforced and probe data for model validation is not warranted. Thus, we used the simulated go-response probabilities for go and no-go trials to validate which parameters are necessary to recapitulate the patterns observed in participants’ behaviour (see Figs 10 and 11 in S1 Appendix). We found that only the winning model that included separate bias parameters for blocks with and without reinforcement could replicate the observed difference in go-response probabilities between reinforced and probe trials (see Figs 3E and 12 in S1 Appendix).

3) Fixed learning rate

For our models we used a fixed learning rate of 0.06. Initially, we wanted to show that the value for the learning rate does not make a difference for our conclusion by conducting the model validation for five other learning rates (and therefore reproducing the artefact). As our main result is the adaptive response bias, we now prove that results of the model comparison are independent of the pre-defined learning rate. Accordingly, we changed it in the methods, as reproduced below (line 603-605):

With this set of learning rates, we fitted all four models and compared them. We found that the bias model performed best, while the temperature model was the worst fitting model, independent of the learning rate (see Section 3.1. in S1 Appendix).

4) Implications of the initial Q-values

While going through the reviewers’ comments we had the impression, that we did not clearly explain the implications of the positive value initialization in the context of the softmax probabilities. Therefore, to clarify, we decided to reword this paragraph (see line 309-317), as reproduced below:

Thus, the initial value estimate is already shifted from zero (dashed line) to higher values (grey line). The difference between the two curves describes the reduced go-response probability for probe trials compared to reinforced trials. This difference is smaller for go stimuli than for no-go stimuli; During the acquisition of values for go stimuli, both curves quickly converge towards 1 (green arrow), while the exact opposite happens for no-go stimuli. These likewise start at a relatively high positive value, but because they are updated in the opposite direction during learning, the difference between the two curves first increases before decreasing again when converging towards -1.

5) Line 230-232

We found and error in the text and corrected it - the baseline model four (not three) free parameters:

The baseline model included four free parameters: one single softmax temperature τ and a bias term b for both block types, one initial value estimate Q_0 for each of the twelve stimuli, and a decay parameter θ.

6) Finally, we made some minor textual modifications to improve the comprehensibility of those passages.

References

1. Jocham G, Brodersen KH, Constantinescu AO, Kahn MC, Ianni AM, Walton ME, et al. Reward-Guided Learning with and without Causal Attribution. Neuron. 2016 Apr;90(1):177–90.

2. Kuchibhotla KV, Hindmarsh Sten T, Papadoyannis ES, Elnozahy S, Fogelson KA, Kumar R, et al. Dissociating task acquisition from expression during learning reveals latent knowledge. Nat Commun. 2019 Dec;10(1):2151.

3. Herzog MH, Fahle M. The role of feedback in learning a vernier discrimination task. Vision Res. 1997 Aug;37(15):2133–41.

4. van Lieshout LLF, Traast IJ, de Lange FP, Cools R. Curiosity or savouring? Information seeking is modulated by both uncertainty and valence. Verguts T, editor. PLOS ONE. 2021 Sep 24;16(9):e0257011.

5. Ritvo VJH, Turk-Browne NB, Norman KA. Nonmonotonic Plasticity: How Memory Retrieval Drives Learning. Trends Cogn Sci. 2019 Sep;23(9):726–42.

6. Luettgau L, Tempelmann C, Kaiser LF, Jocham G. Decisions bias future choices by modifying hippocampal associative memories. Nat Commun. 2020 Dec;11(1):3318.

7. Schönbrodt FD, Wagenmakers EJ. Bayes factor design analysis: Planning for compelling evidence. Psychon Bull Rev. 2018 Feb;25(1):128–42.

8. Guitart-Masip M, Huys QJM, Fuentemilla L, Dayan P, Duzel E, Dolan RJ. Go and no-go learning in reward and punishment: Interactions between affect and effect. NeuroImage. 2012 Aug;62(1):154–66.

9. Hautus MJ. Corrections for extreme proportions and their biasing effects on estimated values of d′. Behav Res Methods Instrum Comput. 1995 Mar;27(1):46–51.
